# Telehealth Use in Pediatric Care during the COVID-19 Pandemic: A Qualitative Study on the Perspectives of Caregivers

**DOI:** 10.3390/children10020311

**Published:** 2023-02-06

**Authors:** Gergana Damianova Kodjebacheva, Charlotte Tang, Faith Groesbeck, Loretta Walker, Jillian Woodworth, Jennifer Schindler-Ruwisch

**Affiliations:** 1 College of Health Sciences, University of Michigan-Flint, Flint, MI 48502, USA; 2 International Institute, University of Michigan, Ann Arbor, MI 48109, USA; 3 College of Innovation and Technology, University of Michigan-Flint, Flint, MI 48502, USA; 4 Department of Occupational Therapy, University of Michigan-Flint, Flint, MI 48502, USA; 5 Marion Peckham Egan School of Nursing & Health Studies, Fairfield University, Fairfield, CT 06834, USA

**Keywords:** children, caregivers, parents, telehealth, pediatric

## Abstract

This qualitative study surveyed caregivers regarding their perspectives on the benefits of, challenges with, and suggestions for improving telehealth during the COVID-19 pandemic. Caregivers who had the responsibility for caring for at least one child aged under 18 years of age in Genesee County, MI, participated. The caregivers were biological parents, stepparents, foster parents, adoptive parents, and guardians. A total of 105 caregivers completed a survey with open-ended questions via Qualtrics. Two independent coders developed themes based on the responses using grounded theory. Participants were primarily biological parents and non-Hispanic White and African Americans. According to the participants, the benefits of telehealth included prevention of exposure to the COVID-19 virus, quality communication with physicians, savings in travel time, and cost-effective methods to receive care. The challenges included a lack of in-person interaction, fear of compromised confidentiality, and the potential for misdiagnosis. Suggestions for improvement by caregivers included increasing access to telehealth for poorer families, offering a media educational campaign to promote telehealth use, and creating a universal platform to share patient information. Future studies may test the effectiveness of interventions such as those suggested by caregivers in this study to improve telehealth.

## 1. Introduction

The United States reported its first case of coronavirus disease 2019 (COVID-19) on 20 January 2020 [1]. The World Health Organization announced the surge of the COVID-19 virus as a global pandemic on 11 March 2020 [1]. The United States then declared a national emergency on 13 March [1]. By April 14, the American Academy of Pediatrics (AAP) published recommendations to increase telehealth use to help limit the spread of the virus [2]. Medicare and Medicaid increased telehealth coverage [3]. The updated September 2021 AAP guidelines strongly urge pediatricians to continue to offer well, acute, and chronic care visits for children through telehealth and complete in-person elements when possible [4]. 

Telehealth is the remote provision of health services and health education [5]. It is the delivery of patient care using telecommunications technology and thus involves the electronic transmission of health care information [5]. Synchronous telehealth involves the use of video and telephone visits when real-time electronic communication or transfer of information occurs [6]. Asynchronous telehealth is defined as the non-real-time transmission of data and can include patient portal messages, remote patient monitoring, and electronic consultations between general practitioners and specialists (eConsults) [3,6].

In pediatric care, telehealth can be delivered through different approaches. In a systematic literature review of 10 randomized-controlled trials in pediatric care, studies described the following [7]. Approaches involved conducting health screening visits for pediatric patients and their caregivers via teleconferencing [8,9], giving educational sessions on medication management for the pediatric patient and the caregiver through teleconferencing [10], and offering video and/or telephone consultations with obesity specialists [11], dieticians/nutritionists [12,13], and mental health therapists [14,15]. Two of the 10 studies involved remote patient monitoring, specifically performing a bilateral smartphone otoscopy for the child at home [16], measuring blood glucose levels at home, and reporting the readings on a mobile application by the caregiver [17]. One of the 10 studies involved the use and storage of photographs in the electronic medical record for dermatology visits [18]. Pediatric telehealth, thus, uses remote technology to exchange health care information and education in health care.

Pediatric telehealth offers both benefits and challenges for caregivers. A caregiver is defined as a person who has the responsibility for caring for a child under 18 years of age and could include biological, step, foster, and adoptive parents and guardians. An important benefit of telehealth use for caregivers is receiving needed care for their children instead of foregoing treatment [19]. Telehealth can be especially advantageous for caregivers of children with chronic health conditions who need frequent follow-up appointments [3,19,20]. Reduced respiratory virus transmission among both caregivers and children is yet another advantage [3]. Other benefits for caregivers include saved costs and time for travel and fewer missed workdays [21]. For children, telehealth contributes to fewer school days missed [21].

In studies with close-ended survey items, challenges with the use of telehealth included problems communicating with the medical provider [22,23,24]. For example, in the study of McCoy et al. (2022), the ability to communicate with the physician was rated higher (mean 4.6) in the in-person group compared to the video consultation group (mean 4.4) (*p* = 0.012) [22]. In the study of Ragamin et al. (2021), 34.7% of caregivers who received in-person consultations were very satisfied compared to 12.1% of caregivers who received remote consultations (*p* < 0.001) [24]. Caregivers who received in-person consultations were significantly more satisfied on the emotional support scale compared to those who received remote consultations (*p* = 0.039) [24].

Many prior studies on telehealth perspectives were conducted in adult patient care rather than pediatrics [25,26,27,28,29] and among pediatric health care providers instead of caregivers of children [30,31]. Limited research is available on caregivers’ perspectives on benefits, challenges, and suggestions related to telehealth by seeking deeper thoughts through open-ended questions. Most prior studies administered surveys with close-ended items only on the benefits and challenges of telehealth without emphasizing suggestions for improvement [22,23,24,32,33,34,35,36,37,38,39,40,41,42].

Caregivers best understand the needs of their children and can therefore contribute to the enhancement of health care by offering their perspectives on the benefits and challenges, and suggestions for improving telehealth. This qualitative study surveyed caregivers to ask their perspectives on the benefits of, challenges with, and suggestions for improving telehealth during the COVID-19 pandemic. All caregivers in this study reported they used any telehealth services at least once during the COVID-19 pandemic.

## 2. Materials and Methods

*Qualitative nature of the study.* The study was primarily qualitative because it used open-ended items where participants could freely type their explanations on the benefits of, challenges with, and suggestions for improving telehealth. The study sought to adhere to the Items in the Standards for Qualitative Research (SRQR) [43].

*Recruitment.* We collected information among caregivers (1 caregiver per family) in Genesee County between October 2020 and September 2021. From this point on, the word “caregiver” is used to describe biological parents, stepparents, foster parents, adoptive parents, and guardians. Flyers describing the study were placed in the Flint’s farmer’s market, pediatrician offices in close proximity to the Flint’s farmer’s market, and the University of Michigan–Flint in Genesee County, MI. Building/office administrators granted permission to place flyers.

A convenience sampling method was used where all interested individuals who qualified based on the responses to the survey were eligible. The iterative process included the continued placing of flyers to increase the number of participants. The flyers were removed once a sample size of at least 100 was reached and when no one completed the survey in the past 5 days.

*Setting*. All three locations (i.e., Flint’s farmer’s market, the pediatrician offices, and the University of Michigan–Flint) were within less than 1 mile of each other. All were located in downtown Flint. Flint is the largest city in Genesee County, MI. The Flint’s farmer’s market is the largest indoor farmer’s market in Genesee County. Since attendees of the Flint’s farmer’s market, the pediatrician office, and the University of Michigan–Flint may tend to be from Genesee County as a whole (rather than only Flint), the following are population estimates from 1 July 2021 on Genesee County, MI to understand the study setting. Persons under 18 accounted for 22.3% of the population [44]. Households with a computer between 2017–2021 were 90.8% of all households, and households with a broadband Internet subscription, under age 65, were 83.7% of all households. The percentage of persons aged 25 years and older with a bachelor’s or higher degree was 22.2%. The percentage of persons living in poverty was 18.3%. The median household income in 2021 dollars was $54,052 in Genesee County, MI [44].

*Ethical approval and matters.* The study received Institutional Review Board (IRB) approval from the University of Michigan–Flint on 11 June 2020. When one opened the link to the survey, the first page included descriptive information regarding the survey with an emphasis that participation was voluntary. The first page also emphasized that the participant could stop the survey at any time or could choose not to answer any question. Caregiver names were deleted once cash gift cards in the amount of $25 for each completed survey were distributed. The names of pediatrician offices are not stated to protect their confidentiality per the IRB approval.

*Survey*. Participants completed surveys from 7 July 2020 until 5 January 2021. The flyers included a link to the Qualtrics survey and a code that could be scanned by one’s mobile telephone for direct access to the survey. The beginning of the survey indicated: “All the questions below are regarding YOUR CHILD’S HEALTH CARE, not about your own health care. If you have more than 1 child, please answer the questions only regarding your youngest child.”

Caregivers were asked regarding any prior use of telehealth for their child through the following close-ended item with a Yes/No response option: “During the COVID-19 pandemic, did you at least once use telehealth (i.e., any of the following: video and/or telephone visits/conversations with the medical provider, electronic medical records, electronic surveys prior to visits and/or electronic check-in, electronic refill of a medication, electronic request for a referral to a specialist, exchange of electronic messages with the medical provider, and other remote/virtual health care/services) for your child?”

The survey then included the following open-ended questions:

- What are the benefits of telehealth, if any, during the coronavirus pandemic?
- What, if anything, keeps you from using telehealth for your child during the coronavirus pandemic?
- If you could design strategies to make telehealth better for the care of your child and/or other children during the COVID-19 pandemic and overall, what suggestions for improving telehealth do you have?

Following the above open-ended items, there were socio-demographic items on the caregiver’s role (i.e., biological parent, stepparent, foster parent, adoptive parent, and guardian), race and ethnicity, relationship status, and level of difficulty living on household income.

*Data processing and analysis*. Once the gift cards were mailed, the name and addresses of participants were deleted. The anonymous survey responses to the close-ended items were transferred into Stata 17 for data coding and analysis. The complete survey responses to open-ended items were obtained from Qualtrics. Since respondents entered their responses, there was no need for transcription.

According to the Standards for Reporting Qualitative Research (SRQR), the process by which themes are identified and developed should be described and should include the names of the researchers involved in data analysis [43]. In addition, according to the SRQR, techniques to enhance the trustworthiness and credibility of data analysis include researchers independently developing themes at first, checking and revising each other’s themes, and keeping an audit trail of the original and revised themes [43].

We analyzed the responses using a grounded theory approach. The rationale for using grounded theory is that grounded theory is a systematic method of collecting and analyzing qualitative data [45]. The goal of the grounded theory approach is to generate themes and subcategories within themes that emerge from the participant responses [45]. The responses to the survey’s open-ended questions were read independently by 2 team members (GK and LW). To reduce subjective assessments by researchers, each of the two members developed themes independently in separate Excel files. The Excel files had columns on Benefits, Challenges, and Suggestions. Once each member completed the development of themes and subcategories, each member read the other member’s findings to determine common themes between the 2 members and themes that each member may have missed/interpreted differently. Analysis was complete when (1) no new themes or subcategories emerged and (2) agreement between members existed through discussion.

## 3. Results

### Survey Results

Most survey participants were White, employed, and either single or married. Most were biological parents (Table 1). All 105 participants used any telehealth at least once during the COVID-19 pandemic according to responses to the Yes/No item: “Have you used telehealth at least once for your child during the COVID-19 pandemic?”

Caregivers described the benefits of telehealth (Table 2). Video visits included the following benefits: minimized exposure to COVID-19, savings in travel time, opportunity to receive quality care, and cost-effective methods to receive care. The use of other types of telehealth, such as email, test results, and electronic survey completion prior to the visit, offered the following benefits: direct communication with physicians and cost-effective, convenient, and autonomous care.

Some participants experienced no challenges with telehealth. For example, participants stated:

○ *“We had a telehealth appointment to receive results and talk with a specialist. I don’t think I would have changed anything. It went very smooth and actually was a nice alternative to sitting around the hospital for hours waiting for the results”*
○ *“I think under the current circumstances all of my child’s healthcare workers have risen to the challenge of telehealth model of treatment;”*
○ *“Nothing has kept me from using it. I use it 3–4 times a week.”*
○ *“I use telehealth and it is pretty straightforward.”*
○ *“They are doing great, and I was so impressed with how fast they switched over to remote.”*

One challenge with video visits was the lack of in-person interaction (Table 3). Specifically, participants believed that in-person discussions could trigger more thoughts and questions. Participants believed they could process information better during in-person visits. Fear of compromised confidentiality and potential misdiagnosis were other challenges described by participants.

The patient portal and electronic messages to communicate with the medical provider also included challenges. Portals used by different healthcare systems made accessing information more difficult compared to the use of a universal system. The use of email for medical discussion left the potential for misinterpreting information both by the physician and caregiver. It could result in a delayed response compared to making a telephone call.

Caregivers offered suggestions to increase the availability of telehealth for all and increase access to telehealth for poorer families and/or those who lack technology (Table 4). Caregivers expressed that there was a need for increased availability of video and telephone visits. They stated that they would consider switching to a different medical provider if telehealth were not available. Caregivers emphasized that if there was no need to have an in-person appointment, then the appointment should be online. Governmental funding should be used to increase access to computers and affordable internet among poorer families.

Caregivers had the following additional suggestions for improvements: provide education on how to use remote care at the medical provider office, offer advertisements and media campaigns, create a universal platform/a single platform to share patient information, and involve young patients in the telehealth process (Table 5). Educational materials and workshops at the provider’s office should explain how to use telehealth. The media campaign should utilize captivating images to promote the use of telehealth. The universal platform should help create consistency among providers in terms of the types of telehealth offered. It should allow for the linkage of information in the same state and across states.

## 4. Discussion

### 4.1. Summary

The benefits of video and/or telephone visits (i.e., synchronous telehealth) included the prevention of COVID-19 virus exposure, the opportunity to receive quality care, savings in travel time, and cost-effective care. The benefits of patient portals and email messages with the medical provider (i.e., asynchronous telehealth) included direct communication with physicians and cost-effective, convenient, and autonomous care.

Despite these benefits, challenges such as lack of in-person interaction and technological difficulties existed. Suggestions for improvement included increasing availability of remote visits, enhancing access for poorer families, offering education on how to use telehealth in the medical provider’s office, offering media campaigns to increase awareness of telehealth, creating a universal platform/a single platform to share patient information, and actively involving young patients (i.e., primarily adolescents) in remote care.

### 4.2. Interpretation of Findings on the Benefits of Telehealth

Similar to this study, in prior research, the strengths of telehealth visits indicated by caregivers using telehealth included time savings [21], convenience [21,23], and protection from COVID-19 [3,23]. The finding in this study that caregivers’ questions were answered to satisfaction during telehealth visits is consistent with studies that found high satisfaction with virtual care [35,36,37,38,39]. Eighty-two percent of parents in a study of 281 parents in a pediatric pulmonary clinic either strongly agreed or agreed that they would use telehealth services again [46]. Future research may need to assess the benefits of telehealth in the physical and mental health outcomes among pediatric patients.

### 4.3. Interpretation of Findings on the Challenges with Telehealth

Caregivers reported a preference for in-person visits because they could develop a better rapport with the medical provider if in the same room. In surveys, both pediatricians [47] and caregivers [48] reported a patient preference for in-person care as a barrier to telehealth use. In a study of two focus groups with caregivers of children on online treatment for pediatric rehabilitation in Israel, investigators identified a theme: “most feel that nothing replaces person-to-person contact that brings really meaningful continuity” [48]. Training for medical students and providers, therefore, on how to build rapport with and earn the trust of the patient and caregivers will be valuable. A 2-hour patient-centered training on virtual visits for medical students included education, role-play sessions with simulated patients, and a group debriefing on how to build rapport and earn trust with patients and families by video [49]. To build rapport and earn trust, the study recommended that medical students and providers use good lighting, look directly at the camera, ensure that the attention is on the patient, request permission from the patient to take notes, and ask direct questions to understand the patient’s emotional state because patient stress can be more difficult to interpret on video [49].

Caregivers expressed a concern that misdiagnosis may occur when using virtual physician appointments in the present study. Virtual visits may be supplemented by home monitoring devices such as blood pressure monitors, pulse oximeters, digital stethoscopes and otoscopes, home spirometry devices, and remote devices to track glucose, capture an electrocardiogram, and conduct sleep studies [50]. There will be a need for additional home monitoring devices or supplemental in-person visits to avoid misdiagnosis. It may be difficult for caregivers to procure these medical devices out of their own pocket. Therefore, we suggest programs expand access to home monitoring devices. Health clinics and hospitals may implement programs that allow patients to loan needed medical devices for use during virtual appointments. To minimize traveling, there should be an option for the patients to receive and return the devices by mail.

Caregivers reported that they were concerned if information exchanged through telehealth was confidential. Similarly, themes identified during two focus groups with caregivers in Israel on online care for pediatric rehabilitation were: “data security issues and need to make sure that interactions are (ethically) compliant” [48]. Assurances need to be provided to caregivers that telehealth services are confidential so they can freely discuss their concerns in the same way as during in-person consultations. In multiple-person households, this confidentiality issue can be particularly challenging when attending virtual appointments at home. Creating an environment in which caregivers and adolescents can freely discuss their concerns may be accomplished by asking caregivers and/or adolescents to move to a private space, asking others to leave the room, and having caregivers and/or adolescents type questions and answers and wear headphones with the provider asking yes-no questions [39].

### 4.4. Interpretation of Findings on the Suggestions to Improve Telehealth

A unique contribution of the current study is the caregivers’ suggestion on the need for the development of telehealth media campaigns and advertisements. A media campaign should create captivating images and spread the word about the existence and benefits of telehealth, according to the caregivers.

Another suggestion by the caregivers was the need for education on how to use telehealth in the medical provider’s office. The American Academy of Pediatrics developed a “Promoting Telehealth Campaign Toolkit” to be used by medical providers [51]. Medical providers can use the toolkit to let young patients and caregivers know that telehealth is an option for medical visits. They can use the toolkit to help families understand the basics of a telehealth visit and how to prepare for one [51].

Although telehealth has become more accessible, disparities in accessibility by sex, residing area (metropolitan vs. nonmetropolitan), income level, and US Census region exist [52,53]. Caregivers, therefore, understandably recommended expanding telehealth for families who live in poverty or lack access to computers. A solution is providing private rooms at local libraries for remote health care. Yet another solution is for local community organizations to offer access to their computers and wi-fi connection for video visits. New programs and policies such as the Connected Care Pilot Program and the Coronavirus Aid, Relief, and Economic Security act seek to allocate funding for expanded access to telehealth and the internet in the United States [54]. Future research should assess the influence of new programs and policies on socio-economic disparities in the use of and satisfaction with telehealth.

Research on adolescents’ opinions related to telehealth is especially limited [55,56,57,58,59,60,61,62,63,64,65]. Caregivers’ suggestions were to actively involve adolescents, such as by providing them with passwords for patient portals, educating them on how to use services, and asking for their ideas on how to improve remote health care.

### 4.5. Generalizability of Findings

The results seek to highlight the lived experiences of the caregivers and suggest areas for attention for further research. The participants may not represent all residents of Genesee County and/or Michigan. According to the population estimates of 1 July 2021, 79.0% of Michigan residents were White, and 14.1% were Black/African American [44]. According to the population estimates of 1 July 2021, 75% of Genesee County residents were White, and 20.3% were Black/African American [44]. In this study, 65.7% were White, and 26.7 were Black/African American. The racial characteristics of our participants may be more representative of Genesee County than Michigan. Participants in this study tended to be of higher socio-economic status with access to health care. Future research should investigate suggestions for improving telehealth among individuals of lower socioeconomic status.

### 4.6. Study Limitations

The limitations of the study included the convenience sampling, small sample size, lack of categorization on the challenges with telehealth use by insurance status and type, and lack of surveys among children and adolescents on their needs. Future studies may assess differences in satisfaction with telehealth use by insurance status and type. They may survey children and adolescents on how remote health care may better serve them.

The level of participation (such as the number of respondents compared to the total number and characteristics of patients at the pediatrician’s office) was not ascertained. Another limitation is that the survey did not ask participants about the frequency of telehealth use, such as whether caregivers were frequent users or first-time users. They may have discussed telehealth services they primarily used.

Qualitative investigations have the purpose of collecting thoughts by asking open-ended questions. In qualitative investigations, it is recommended to ask broad questions so that new and unexpected ideas emerge based on people’s experiences. Future research may focus on administering surveys with close-ended items on perspectives in a larger sample size of caregivers recruited through random sampling.

The quotes on caregivers’ familiarity with telehealth tended to confirm the close-ended survey response that participants used some types of telehealth services. Still, a limitation was that triangulation of sources/methods was not conducted; a future study may conduct both surveys and one-to-one interviews to understand the benefits of, challenges with, and suggestions to improve telehealth.

## 5. Conclusions

There is a need for the development of a media campaign to increase awareness of telehealth, according to caregivers. The media campaign should create captivating images and spread the word about telehealth. A media campaign may highlight both the benefits and challenges of telehealth so that parents can decide the best option for health care, depending on their needs. Our study identified a variety of benefits of telehealth, including the continuity of care. Interventions that enhance telehealth may reduce health care costs by encouraging regular health care, improving medication management, increasing access to regular health care by different socio-economic groups, and ultimately improving health outcomes.

## Figures and Tables

**Table 1:** Characteristics of caregivers who completed a survey regarding the use of telehealth for their child during the COVID-19 pandemic.

	Study Population, N = 105
**Relationship to the child answering questions about, n (%)**	
Biological parent	91 (86.7)
Stepparent	4 (3.8)
Foster parent	1 (1.0)
Adoptive parent	2 (1.9)
Guardian	7 (6.7)
**Age, n (%)**	
18–30	25 (23.8)
31–40	26 (24.8)
41–50	18 (17.1)
51–60	2 (1.9)
Did not answer	34 (32.4)
**Race, n (%)**	
White/Caucasian	69 (65.7)
Black/African American	28 (26.7)
American Indian/Native American	1 (1.9)
Alaskan Native	0 (0)
Asian	7 (5.7)
Native Hawaiian	0 (0)
Pacific Islander	0 (0)
**Ethnicity, n (%)**	
Hispanic/Latino	6 (5.7)
Arab	3 (2.9)
Neither of these	96 (91.4)
**Relationship status, n (%)**	
Single–never married	24 (22.9)
Married	50 (47.6)
Other committed relationship	17 (16.2)
Separated	2 (1.9)
Widowed	1 (1.0)
Divorced	11 (10.5)
**Difficulty living on household income, n (%)**	
Not at all difficult	32 (30.5)
Somewhat difficult	50 (47.6)
Difficult	15 (14.3)
Very difficult	6 (5.7)
Extremely difficult	2 (1.9)

**Table 2:** Telehealth benefits, along with representative quotes by the caregivers.

		Study Population, N = 105
**Video and telephone visits** Minimizes exposure to disease and the COVID-19 virus while receiving healthcare ○ *Participant: “By keeping kids at home and having online visits for doctor appointments. Only bring kids in when needed to keep kids safer from being exposed to dangerous virus.”* ○ *Participant: “I think it is necessary to provide health information technology for everyone during this crisis, to avoid COVID-19.”* ○ *Participant: “It is something that is very useful before the pandemic and of the utmost importance during the pandemic.”* ○ *Participant: “It is healthier if a person stays at home.”* ○ *Participant: “Safer environment for healthcare needs; no mask needed.”* Eliminates need to travel and take time off work ○ *Participant: “I would have had to drive my daughter 40 minutes one way and 40 minutes back for her health appointments. The telehealth made it so that I did not have to leave the house or request time off work at all.”* Provides convenient and quality care/information ○ *Participant: “We had a zoom meeting with a surgeon and it was great. The doctor was informative and was able to give us great information.”* ○ *Participant: “I was grateful for a quick and easy video visit when child got hurt. I am glad this tech exists.”* Is a cost-effective method to receive care and directly communicate with physicians during video visits ○ *Participant: “Some places have a reduced fee for online visits which is helpful because we have a high-deductible plan.”* **Email, test results, electronic survey completion prior to visit** Offers convenience, autonomy, faster service ○ *Participant: “I can use email at any time I want to.”* ○ *Participant: “Communication, through emails, there is always a way to reach out to someone.”* ○ *Participant: “Appreciated the ability to email my child’s physician and get a response so quickly without having to call or attempt to reach someone.”* ○ *Participant: “We can access his (the child’s) results faster.”* Is a cost-effective method to receive care and directly communicate with physicians through email ○ *Participant: “Just being able to send a quick message and hear back from the pediatrician without, one, having to pay for an appointment and, two, go in, was really helpful.”* Shortens waiting time for in-person visits by electronically completing patient paperwork in advance ○ *Participant: “Filling out questionnaires prior to going in is very useful. It lessens the time you’re in the waiting or exam room.”*		

**Table 3:** Telehealth challenges, along with representative quotes by the caregivers.

		Study Population, N = 105
**Video and telephone visits** Lack of in-person interaction/preference for in-person visits ○ *Participant: “Children are depersonalized when they’re in a video call…it’s like a 50% appointment.”* ○ *Participant: “In-person visits are better. You lose the relationship or sense of empathy when you’re not in the same room.”* ○ *Participant: “I believe face-to-face is the best care for well-visits.”* ○ *Participant: “I don’t think telehealth would have worked for a well visit.”* ○ *Participant: “I prefer face to face as long as the office follows safety precautions.”* ○ *Participant: “Given the issues my daughter has had, the in-person care has been more of what was necessary.”* Technological problems ○ *Participant: “We just couldn’t get it to work. It was frustrating to say the least.”* ○ *Participant: “Televisit technology needs to be more reliable. Our personal experience took several attempts to connect. My child had a fever and a head-to-toe rash, and the doctor could not see things because the app ran so poorly.”* ○ *Participant: “The endocrinologist for my son was a disaster as telehealth. The appointment had the worse connection. Worthless appointment.”* ○ *Participant: “Connection issues, trouble accessing records (keeps me from using it).”* Fear of compromised confidentiality ○ *Participant: “I guess the worry was if your information is still confidential.”* ○ *Participant: “Awareness and personal records security (keeps me from using telehealth).”* Difficulty in understanding/receiving information. Easier/less stressful to process and understand information given in person ○ *Participant: “The information is still there but I receive it better in person.”* ○ *Participant: “The stress from COVID-19, I do not want to continue researching and using electronic devices.”* Potential misdiagnosis due to camera angle, lighting, etc. ○ *Participant: “It could leave room for error or a misdiagnosis because something might look like something else (when) you’re not looking at it up close and personal.”* **Patient portal, email, appointment scheduling** Problems with multiple portals or logins ○ *Participant: “The different portals do not interface.”* ○ *Participant: “We should still be able to login to both charts as we are 2 separate individuals without continued access issues and blocking access frequently.”* ○ *Participant: “A user-friendly app would be great that’s easy to access without a million passwords to get into it.”* Potential for misinterpreting email messages both by the physician and patient ○ *Participant: “When you are typing things out, things can be misinterpreted.”* Impersonal communication ○ *Participant: “It didn’t feel very personable, because it is usually a text message or just like a standard written email.”* Slow response time after sending an email to the physician ○ *Participant: “If I call, they will help me at that moment versus if I use health technology, it probably would take a couple of days.”* Online availability did not reflect all days/times available for scheduling appointments ○ *Participant: “if we go online to try and schedule an appointment: there is less availability then if we were to actually call. Then there is a little more availability.”*		

**Table 4:** Suggestions for increasing availability of and access to telehealth along with representative quotes by the caregivers.

		Study Population, N = 105
**Increase availability of telehealth** Make telehealth more widely available ○ *Participant: “I think all doctors should give the option of having an appointment through video or a telephone. Some don’t, and then the kids have to wait months to get in for an appointment.”* ○ *Participant: “They could offer more telehealth appointments.”* ○ *Participant: “More tele-health visits for things that don’t need to be seen in person.”* ○ *Participant: “Give the option for televisits instead of in person.”* ○ *Participant: “To better meet the needs of children, we need to have the ability to see medical records online or make appointments online.”* ○ *Participant: “Definitely make electronic health more accessible by adding a portal.”* ○ *Participant: “More options for those with minors, dependents; online dedicated team for pediatric and family care, follow-up, video visits, education.”* ○ *Participant: “Send more alerts and reminders for vaccinations and well child visits.”* ○ *Participant: “I think it is essential to offer health information technology, especially during the crisis. Part of the reason I am considering switching pediatricians is the limited health information technology.”* ○ *Participant: “Explore the evolving technology because it seems the virus will not be disappearing so they need to offer more.”* ○ *Participant: “Have phone or video appointments when shots/vaccines aren’t necessary.”* ○ *Participant: “Start setting up most appointments online and only have kids come in for tests or vaccinations. If it can be done online, then it should be done online. Having things like speech therapy or therapy that can be done online be online.”* **Increase access to telehealth for poorer families and/or those who lack technology** Provide government funding or other support to make telehealth, such as computers and the internet, accessible for lower-income families ○ *Participant: “I am a very fortunate person. But there are people that do not have the technology. If only there was grants funding to give a new mom a phone and a portal to have her take care of her child.”* *Participant:* “*I’d probably do government funding to improve telehealth. I think that the government would support that*”. ○ *Participant: “State aid will probably help. And that could fall under the health department helping fund.”* ○ *Participant: “Another thing for consideration are those families who do not have access to the appropriate technology in their home to give them the means.”* ○ *Participant: “Just make sure everyone knows about it. I only found out on the website. Some may not afford a computer to see it.”* ○ *Participant: “Think of ways to help those who don’t have technology.”* ○ *Participant: “Offers hubs that are available for people without technology.”*		

**Table 5:** Suggestions for offering education, advertisements and media campaigns, a universal platform for sharing patient education, and opportunities for young people to be involved in telehealth, along with representative quotes by the caregivers.

		Study Population, N = 105
**Offer education to the caregiver and/or young patient, usually through the medical provider’s office** Create/provide instructional materials or education; create an education platform/program ○ *Participant: “I believe there are ways to access the patient chart through electronic means, I have never been instructed on how to do so. I think better education on how to use health information technology would be advantageous.”* ○ *Participant: “Making sure people are able to access the sites and have knowledge of how to use them.”* ○ *Participant: “Explain telehealth better to the parent and our teens.”* ○ *Participant: “Send out information on how to access it. There is poor access to education/multiple hoops to jump through.”* ○ *Participant: “Please offer a variety of technology options at staged/staggered intervals with education so that the novice users can get acquainted with things and gradually increase the complexity of their access as their expertise grows.”* ○ *Participant: “Make the user interface very user-friendly and put together great tutorials for non-computer using individuals.”* ○ *Participant: “Make it very easy to use and provide easy to understand instructions for the whole family including the children.”* ○ *Participant: “Provide very clear concise steps for creating online profiles, suggest they save their username and password somewhere. Perhaps they could also include screenshots of the step-by-step process for logging in and navigating or a video tutorial.”* ○ *Participant: “Give the education and webinars to educate them (the caregivers and patients).”* ○ *Participant: “It is worth a try. Maybe doing a walkthrough or practice visit so people understand is not bad.”* ○ *Participant: “Tutorials and examples could be used to help people learn to use health tech info.”* ○ *Participant: “Offer instructions to those who may struggle with the technology.”* ○ *Participant: “Clear and concise directions/step-by-step aides.”* ○ *Participant: “Zoom instruction meetings hosted by healthcare providers to help patients understand the value and how to use the technology.”* **Offer advertisements and media campaigns** Use commercials/advertisements to increase awareness of the existence and benefits of telehealth ○ *Participant:“Use ads on social media because that’s the audience you want.”* ○ *Participant: “I think commercials are great. Our phones listen to everything so maybe a pop-up advertisement on Facebook and Instagram. People are attracted to images.”* ○ *Participant: “Ad in Facebook and Twitter.”* ○ *Participant: “Knowing the benefits to it, knowing what it is, I don’t think a lot of people know what it is. So advertising what it is and advertising the benefits.”* ○ *Participant: “I think the advertising and making people aware that you offer these services is important. I would likely use them if I knew they were available.”* ○ *Participants: “Advertise it and make it easily accessible. People working during this crisis have so many things to worry about and don’t know about it (telehealth).”* Use media campaigns to create captivating images and spread the word about telehealth ○ *Participant: “Spread more awareness of it and the process in the media to make it [telehealth] appear less intimidating.”* ○ *Participant: “I think a media campaign to get the word out would be great so that the people of the community know about it. When the community knows about it [telehealth], we will have a vote and get more funding for it.”* ○ *Participant: “I would encourage them to promote it in the media so that I could use it.”* ○ *Participant: “Promote it and make the services more well known in the media.”* **Offer a universal platform/a single platform to share patient information** Provide a universal platform/a single platform to share information both in the same states and across states ○ *Participant: “A universal platform to gather information to share results and recommendations so our doctors could communicate to provide better overall healthcare.”* ○ *Participant: “Pediatricians can practice medicine across state lines so that people can access the best care.”* ○ *Participant: “It’d be nice for me to be able to gather all the information in one universal system.”* ○ *Participant: Set up one portal with info on all my child’s upcoming tests.”* **Offer young patients with opportunities to use technology and to suggest how to improve telehealth** Make signing-in to technology user-friendly for young patients ○ *Participant: “Make the technology more child-friendly to sign in. My children struggle to get logged into scheduled appointment without me.”* Ask young patients for their suggestions on how to improve telehealth ○ *Participant: “Why not ask kids what they would like? My child has experience seeing the psychologist through technology.”*		

## Data Availability

The data presented in this study are available on request from the corresponding author. The data are not publicly available due to ethical approval guidelines.
